# “If They Are Not Labeled, How Do We Know What’s Risky and What’s Not?”: Assessing Information on Brominated Flame Retardants in Children’s Plastic Products in South Africa

**DOI:** 10.1177/11786302261435748

**Published:** 2026-04-03

**Authors:** Rebecca Mlelwa, Hanna-Andrea Rother

**Affiliations:** 1 Environmental Health Division, School of Public Health, University of Cape Town, Observatory, Cape Town, South Africa

**Keywords:** chemicals-in-products information, children’s products, POPs, Global Framework on Chemicals

## Abstract

**Background:**

Global literature indicates that polybrominated diphenyl ethers (PBDEs) and hexabromocyclododecane (HBCDD), types of brominated flame retardants (BFRs), are increasingly found in children’s plastic products. These products are widely sold in South Africa. PBDEs and HBCDD are persistent organic pollutants (POPs) banned under the Stockholm Convention due to their severe health impacts, including endocrine disruption, neurotoxicity, and cancer. Information about chemicals present in these plastic products is necessary to inform risk reduction measures.

**Methods:**

This study assessed the availability and accessibility of information on POP-BFRs in children’s plastic products in South Africa to enhance stakeholders’ capacity to implement risk reduction measures. The study was guided by the chemicals-in-products (CiP) information-sharing framework developed by the United Nations Environment Programme (UNEP) in 2015. Data collection involved in-depth interviews and an online survey. South African stakeholders participated, including those involved in manufacturing, regulation, and advocacy (n = 10) and consumers (n = 44). Data was analyzed thematically using NVivo.

**Results:**

CiP information on POP-BFRs in children’s plastic products was largely unavailable. Regulatory and advocacy stakeholders had limited general information on POP-BFRs and the associated risks. Additionally, they had limited access to POP-BFRs information, while consumers had none.

**Conclusion:**

National and international coordinated actions are necessary to close the POP-BFRs information gaps. The South African government should develop and enforce an overarching chemical legislation, establish a national chemical register, and require full disclosure of CiP. UNEP should establish a global standardized system for CiP information under the 2023 Global Framework on Chemicals.

## Plain Language Summary

If they are not labeled, how do we know what’s risky and what not? - Assessing information on brominated flame retardants in children’s plastic products in South Africa

**Why was the study done?** The United Nations Environmental Programme (UNEP) emphasizes the importance of providing information about hazardous chemicals in consumer products. This information helps stakeholders make informed decisions and take steps to reduce risks. Brominated flame retardants, which are hazardous chemicals, are increasingly found in children’s plastic products. Children are the most vulnerable to these chemicals’ effects. However, it is unclear whether the necessary information about these chemicals is available and accessible to key stakeholders to help them implement risk reduction measures to protect children.

**What did the researchers do?** The researchers gathered data from 54 South African stakeholders to better understand the availability and accessibility of information on brominated flame retardants in children’s products, enabling us to develop policy recommendations to fill existing gaps.

**What did the researchers find?** Information about the presence of the brominated flame retardants in children was largely unavailable. Law enforcement and advocacy stakeholders had limited access to general information about these chemicals, and consumers had none.

**What do the findings mean?** This study has identified key gaps that should be the focus of future policies, both nationally and internationally, to enhance the availability and accessibility of information about hazardous chemicals, such as brominated flame retardants, in daily-use products. These include creating a comprehensive national chemical law that requires the manufacturing industry to be fully transparent about product chemical content. Additionally, UNEP should develop a standard format for disclosing such chemical information.

## Introduction

Even though chemicals offer some benefits, their negative health impacts have long been recognized.^1,2^ Various chemicals, including fragrances, preservatives, colorants, and flame retardants, are present in consumer products. Many of these chemicals pose significant exposure risks because consumers encounter products daily.^3^ Chemicals can enter the human body through various pathways and routes, depending on how they are applied and the duration of the product’s use.^1^ The broad availability of products containing hazardous chemicals heightens the likelihood of exposure risks more broadly. While each chemical presents different health risks,^4^ exposure to a mixture of chemicals - as they often appear in consumer products - may result in synergistic combined effects.^5-7^ The effects of exposure to a mixture of chemicals are less well understood; therefore, exposure should be prevented and minimized.

Significant concerns exist regarding hazardous chemicals in products used by and intended for at-risk populations, particularly children. This is due to children’s activity patterns and behaviors, such as hand-to-mouth interactions, which significantly increase their exposure to chemicals compared to adults.^8^ Some key chemicals of concern in children’s products include hazardous flame retardants,^9^ heavy metals like lead, mercury, cadmium, and arsenic,^10-13^ phthalates,^14,15^ and per-and polyfluoroalkyl substances.^16^ This research focused on brominated flame retardants (BFRs).

Two types of BFRs, polybrominated diphenyl ethers (PBDEs) and hexabromocyclododecanes (HBCDD), are increasingly found in products intended for children.^17^ Research by Miller et al detected PBDEs in diaper (nappy) changing kits, vinyl baby bibs, and children’s plastic costumes in the United States.^18^ Another recent study in the United States found PBDEs and HBCDD in toys, hair accessories, and food service ware.^19^ Similarly, other researchers have detected PBDEs and HBCDD in a wide range of toys and hair accessories across the European Union and the United Kingdom.^20-22^ Studies conducted in Asian countries found PBDEs in children’s playmats in China,^23^ toys and hair accessories in Japan, and several other countries.^24^ PBDEs and HBCDD have also been found in toys and other children’s products in 17 African countries, including South Africa.^25^ Products, such as toys, are mainly manufactured in China and are widely sold in the South African market.^26^ The diverse range of these plastic childcare products and toys is referred to herein as “children’s plastic products.”

The presence of PBDEs and HBCDD in children’s plastic products is of critical concern for 2 main reasons. Firstly, the 2 groups of chemicals are associated with severe health impacts such as endocrine disruption, neurotoxic effects, and cancer.^9,27-29^ Additionally, they are also classified as persistent organic pollutants (POPs) under the Stockholm Convention, which led to the ban on the production and functional use of PBDEs in the late 2000s^30^ and the HBCDD in 2017^31^ to protect humans and the environment.

Secondly, unlike chemicals such as phthalates that are intentionally added substances (IAS) to plastics, POP-BFRs are non-intentionally added substances (NIAS)^3,32^ in children’s products. They are often found in levels below the concentrations required to impart flame retardancy, hence, regarded as contaminants.^17^ Historically, these hazardous substances were intentionally added to plastic housings of electronic equipment to meet fire safety regulations.^33^ As electronic devices reach the end of their life, their plastic casings are then recycled into new products to minimize waste generation in line with the circular economy.^34^ Researchers believe that inefficient recycling methods and poor control of electrical and electronic equipment waste (WEEE) recycling have led to the transfer of legacy POP-BFRs into recycled products and widespread exposures.^35^ Therefore, information about their presence in products is necessary to inform risk reduction measures.

### Background to the Approach for CiP Information Generation and Sharing

Chemicals-in-products (CiP) information refers to a range of data intended to describe IAS and NIAS contained in a product or their absence, depending on whether there is a set threshold.^32,36^ The information may include chemical names, potential hazards, and details on the safe use and end-of-life handling of products.^37^ Other accompanying details, such as product manufacturer and supplier identification, may also be included in CiP information.^38^ This information is vital for various stakeholders involved in all stages of the product life cycle.

In 2006, the United Nations Environment Program (UNEP) held the first International Conference on Chemicals Management, where the Strategic Approach to International Chemicals Management (SAICM) was adopted as a voluntary framework for chemical management throughout the life cycle.^39^ The issue of CiP information was first recognized as a critical global policy concern during the 2006 conference, and therefore, one of SAICM’s primary objectives was to ensure that “*information about chemicals, including chemicals in products, is available, accessible, user-friendly, adequate and appropriate to the needs of all stakeholders*” (p. 15).^39^

To achieve this objective, UNEP launched a CiP program within the framework of SAICM in 2015. This non-binding program aimed to accelerate the generation and sharing of information regarding chemicals in products to ensure availability and access.^40^ This milestone culminated from prior global surveys that assessed the stakeholders’ information needs, information communication systems, gaps, and necessary actions to promote CiP information flow across 4 priority sectors. The 4 sectors surveyed were toys,^41^ electronics,^42^ building materials,^43^ and textiles.^44^ The findings from those surveys informed UNEP’s guidelines for CiP information generation and sharing.^36^ The guideline includes suggested activities for stakeholders to undertake to meet the CiP information objectives.

As SAICM did not achieve its goal of sound chemical management by its targeted year 2020, its successor, the new Global Framework on Chemicals – For a Planet Free of Harm from Chemicals and Waste (GFC), was adopted during the fifth International Conference on Chemicals Management in September 2023.^45^ Strategic Objective B of the new GFC emphasizes the need for the generation and sharing of CiP information among stakeholders to facilitate informed actions and decisions for effective chemical management by 2030.^45^ Specifically, targets B2, B3, and B7 of the GFC mirror the UNEP’s CiP program, which aims to ensure that relevant data and information on IAS and NIAS in products are publicly available and accessible to all stakeholders.

### Importance of CiP Information in Risk Reduction

Various stakeholders involved in the product’s life cycle have specific interests in CiP information for IAS and NIAS, depending on their needs and literacy levels. For example, product manufacturers require information about the chemical content of raw materials to determine their suitability for use. Waste managers need CiP information to inform their decisions on options for the disposal and recycling of materials. Non-governmental organizations (NGOs) need CiP information for advocacy to influence policies, raise awareness, and protect consumer interests. Consumers, on the other hand, need to know whether products are free from hazardous chemicals, which IAS and NIAS are present to inform their purchasing decision, and how they handle the product. Additionally, government agencies require data on chemical content and their hazard properties to conduct risk assessments and legal enforcement.^37^ Data obtained from risk assessment are then transformed into actionable risk reduction strategies^46^ and non-technical language for public awareness-raising.^47^ These risk reduction decisions are made during every stage of the product life cycle.^48,49^ Therefore, CiP information must be readily available and accessible throughout the product’s life cycle. Also, it should contain relevant details for each stakeholder to apply in their context.

CiP information generation and sharing are the first steps toward achieving its availability and accessibility. In the first stage of a product’s life cycle, the upstream actors, specifically the raw materials and product manufacturers, are primarily responsible for identifying, testing, and documenting the IAS as well as the NIAS in their products and then sharing this information with other stakeholders (eg, retailers and consumers). In cases where CiP information is lacking from manufacturers, the government can generate information and data through product surveillance and monitoring activities and utilize this data for risk assessments, regulatory purposes, and to raise public awareness. The outcomes of generating and sharing CiP information are its availability, that is, the existence of relevant information in the supply chain, and accessibility, meaning it can be obtained and understood by those who need it.

### Study Aims and Conceptual Framework

The increasing evidence of legacy POP-BFRs in children’s plastic products worldwide highlights the need for CiP information about the NIAS to inform appropriate risk reduction measures to safeguard children. At the global level, there is generally a lack of comprehensive CiP information due to a lack of legal mandates for transparency and inefficient information systems.^32,50^ In South Africa, in particular, there is no comprehensive chemical legislation, no legal requirement for full disclosure of CiP information, and no chemical register exists.^51^ As a result, there is no publicly available database for the products and chemicals contained within them. Hence, it remains unclear whether the relevant CiP information regarding POP-BFRs in children’s plastic products is available and accessible to all relevant stakeholders in South Africa to inform their risk reduction decisions.

Therefore, our study aimed to address this knowledge gap by assessing the availability and accessibility of CiP information related to POP-BFRs throughout the life cycle of children’s plastic products in South Africa. This is critical to strengthen stakeholders’ capacity for risk reduction. The main research question was: “What information is available regarding POP-BFRs in children’s plastic products, and how is it shared and accessed by stakeholders involved in the products’ life cycle in South Africa?’

**Figure 1.**
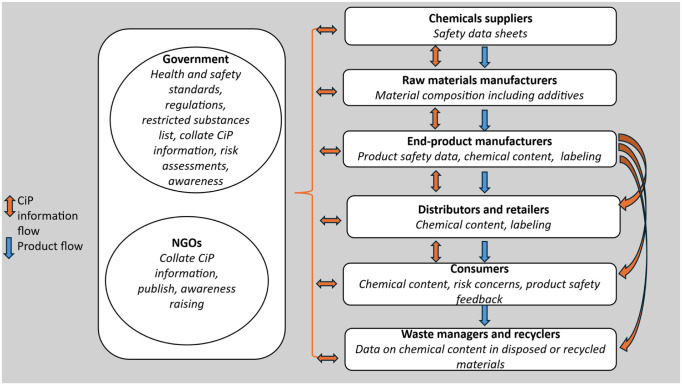
A conceptual framework for assessing availability and stakeholder access to CiP information. *Source*: Adapted and modified from UNEP’s CiP program guideline.^36^

We utilized UNEP’s CiP program guideline^36^ to develop a conceptual framework for this study (Figure 1). We chose to use UNEP’s CiP program guideline because it is the only international guideline providing a framework for achieving the previous SAICM and new GFC goals regarding generating and sharing CiP information. Additionally, it serves as a framework for assessing progress toward making CiP information available and accessible to all relevant stakeholders.

The conceptual framework (Figure 1) outlines a basic model for generating and sharing CiP information, encompassing essential elements such as various groups of key stakeholders, CiP information generated or required at each stage of the product life cycle, information exchange tools, and the flow of information through stakeholders’ engagement. These elements informed our target study population, the variables assessed in the study, and the content of the data collection tools. The conceptual framework was also used as a guide for data analysis. This study is part of a broader investigation into managing health risks posed by POP-BFRs in South African children’s plastic products.

## Methods

### Study Design

Qualitative research methods were employed to gather insights from South African stakeholders regarding CiP information on POP-BFRs in children’s plastic products and the availability and accessibility of this information. Qualitative methods are appropriate for collecting detailed information by capturing individuals’ perceptions and experiences using open-ended dialogues.^52^ Engaging stakeholders in open-ended discussions aimed to gather deeper insights and perspectives, often not captured through quantitative measurements.

### Study Population and Participant Recruitment

Based on the conceptual framework (Figure 1), the study population comprised stakeholders central to generating, sharing, and using CiP information. Six stakeholder groups were identified: government regulatory bodies, NGOs, the chemical and products manufacturing industry, the retail industry, waste management, and consumers. A purposive sampling strategy was utilized to select the study participants from the first 5 groups. The selection process followed a systematic approach. First, a structured online search was conducted using keywords such as “children’s plastic products,” “chemical management,” and “product manufacturing,” focusing on literature published online up to 2023. Second, relevant documents were reviewed, including government reports, organizational websites, and policy and regulatory documents. Third, the research team engaged in iterative discussions and brainstorming to validate and refine the list of stakeholders based on their documented involvement in the product life cycle. The identified stakeholders were then compiled into a database with names, roles in the product lifecycle, and contact information. Afterward, invitation emails were sent to 36 stakeholders across the first 5 groups, along with a study information sheet and an informed consent form. Of these, 10 stakeholders agreed to participate in the study by completing and returning the consent form.

For the consumer group, a convenience sampling method was employed, targeting staff and students at the University of Cape Town (UCT) who were parents or caretakers of children aged 0 to 8 years as potential buyers of plastic childcare products and toys. Before recruitment, permission was obtained from UCT’s Departments of Student Affairs (DSA) and Human Resources (HR). After permission was granted, the DSA and HR departments distributed the invitation to over 25 000 individuals. A total of 44 agreed to participate in the study. Table 1 presents the stakeholder recruitment process, including their role in CiP information.

**Table 1.** Study Participants Recruitment.

Stakeholder group	Role in CiP information	#Contacted	#Agreed to participate
Government	Regulation and information generation	15	5
NGOs	Advocacy and accountability	2	2
Manufacturing industry (chemicals, materials, and products manufacturers)	Primary information generation	7	2
Retail industry	Sector-specific information generation and users	4	0
Waste management industry	Sector-specific information generation and users	11	1
Consumers	End-users of information	25 000	44

Thereafter, participants were categorized into two broad groups: those responsible for generating and sharing CiP information (industry-regulation-advocacy, n = 10) and the consumers (n = 44), who are the end users of CiP information and whose health is to be protected. The industry-regulation-advocacy group comprised 60% males (n = 6) and 40% females (n = 4). The majority were government officials (50%), followed by NGO representatives (20%). Ten percent represented the industry sector involved in flame retardant manufacturing, plastics manufacturing, waste management, and recycling.

The consumer group (Table 2) comprised 79.5% females and 20.5% males. The most significant proportion of participants (47.5%) fell within the 36 to 44 age group, followed by those aged 26 to 35 (40%). A small percentage were 19 to 25 years old (6.8%) or 44 years and older (4.5%).

**Table 2.** Characteristics of the Consumer Group (n = 44).

Characteristics	Frequency (%)
Gender	
Male	9 (20.5%)
Female	35 (79.5%)
Age group	
19-25 y	3 (6.8%)
26-35 y	18 (40.9%)
36-44 y	21 (47.7%)
44+ y	2 (4.5%)
Education level	
Undergraduate	6 (13.6%)
Postgraduate	38 (86.4%)
Participant type	
Students	39 (88.6%)
Staff members	5 (11.4)
Number of children	
1 Child	23 (52.3%)
2 Children	14 (31.8%)
3 or more children	7 (15.9%)

Regarding education level, most held postgraduate degrees (86.4%), while 13.6% had undergraduate degrees. Most participants were predominantly students (88.6%), with staff members making up 11.4% of the consumer group. Additionally, 52.3% had 1 child, 31.8% had 2 children, and 15.9% had 3 or more children.

Once recruitment was completed, each participant was assigned a unique identity number to ensure confidentiality. It is important to note that this study population consisted only of stakeholders who accepted the invitation and consented to participate. This sample does not necessarily represent all potential stakeholders across the entire product life cycle.

### Data Collection

Data collection took place between May 2020 and February 2021. In-depth interviews were conducted with participants in the industry-regulation-advocacy group (n = 10) and a subset of 5 participants (n = 5) from the consumer group. The remaining consumer group participants (n = 39) completed an online qualitative survey. The shift to an online qualitative survey was necessitated by COVID-19 lockdown regulations at UCT, which restricted in-depth interviews with students and staff members to preserve their time for online teaching and learning. Only online surveys were allowed. Therefore, combining the 2 methods allowed the effective inclusion of all participant groups in the study.

The in-depth interview guides consisted of open-ended questions divided into 4 sections: (1) Introduction, (2) Perceptions and Risk Reduction Measures, (3) CiP Information, and (4) Conclusion. The CiP information questions (section 4) were developed based on the UNEP’s CiP program guideline^36^ and the conceptual framework underpinning this study (Figure 1). The questions explored the availability of pertinent CiP information on POP-BFRs in children’s plastic products at each stage of the product life cycle. They also explored stakeholders’ activities in generating, sharing, and accessing the information and the channels utilized for these activities. As described in the UNEP’s CiP program guideline, stakeholders have specific information needs and different roles related to CiP information. Therefore, separate interview guides were developed for each stakeholder in the product life cycle, including chemical and product manufacturers and retailers, government officials, consumers, waste managers, and NGOs.

The in-depth interviews were conducted via Zoom or Microsoft Teams, depending on the participant’s preference. They were semi-structured to encourage free-flowing discussions with participants. Based on participant responses, follow-up questions were asked to explore the responses further and verify whether participants understood the questions or required clarification. Participants were given ample time after each question to respond. The researcher continually reflected on how each session went and what could be improved, such as which questions to ask first, how to ask them, and how to enhance their interviewing skills. The interviews lasted approximately 40 to 60 minutes and were recorded with the participants’ permission.

After the UCT’s research restriction notice, the consumer interview guide was transformed into a qualitative survey. A few closed-ended questions were added to the survey, maintaining a similar structure to the interview guide. The online survey was administered using a Google Form and distributed to participants via email. Responses were exported into a Google Sheet and downloaded in to Microsoft Excel for analysis. The study’s data collection tools are available upon request.

### Data Analysis

The in-depth interview recordings were transcribed verbatim by a professional transcriber. The transcripts and survey files were imported into a qualitative data management software, NVivo 14, for analysis. The qualitative data were analyzed thematically^53^ using inductive and deductive approaches.^54,55^ A thematic analysis followed 6 steps: data familiarization, generating initial codes, searching for themes, reviewing themes, defining and naming themes, and writing the report.^53^

After importing the files into NVivo, the survey responses and interview transcripts were read repeatedly while listening to the audio recording to ensure the accuracy of the transcripts and to familiarize with the entire data set. A series of codes was generated from reading the data (inductively) and from the study’s conceptual framework (Figure 1; deductively). Examples of codes included “regulation,” “safety warning,” “product labels,” and “internet.” Segments of text were highlighted, and codes summarizing the main idea were assigned. Similar ideas that emerged in subsequent files were coded with previously created codes. New codes were generated to capture new ideas. This coding approach continued until all files were coded. Afterward, the codes were reviewed and grouped into initial themes as interpretative stories, guided by the study’s conceptual framework (Figure 1) and research questions.^56^ This iterative process facilitated further review of the initial themes and development of final themes and sub-themes. Additionally, closed-ended questions and descriptive statistics of participants’ demographic data were analyzed using the statistical software SPSS.

## Results

### Study Participants

Data were obtained from 54 stakeholders, divided into the industry-regulation-advocacy group (n = 10) and the consumer group (n = 44).

### Thematic Analysis

Five major themes and sub-themes related to the availability and accessibility of CiP information on POP-BFRs in children’s plastic products were identified. The first theme, *existing information on POP-BFRs*, explores the information reported by industry-regulation-advocacy group participants. The second theme, *gaps in POP-BFR information flow*, explores what participants in both groups experienced as missing or a communication breakdown. The third, *barriers to generating and sharing POP-BFRs information*, includes challenges faced by participants in the industry-regulation-advocacy group. The fourth theme, *consumers’ insights into CiP information access and sources*, explores how consumers seek and access CiP information and the challenges that limit their access to information. The fifth theme, *optimizing POP-BFRs information availability and accessibility*, explores participants’ insights on improving the availability and accessibility of pertinent CiP information. Table 3 links the 5 themes and sub-themes to 3 elements of the study’s conceptual framework (Figure 1)

**Table 3.** Alignment of Study Themes With Conceptual Framework Elements.

Conceptual framework elements	Pertinent CiP information	CiP information generation, exchange, and flow	CiP information access
Themes and subthemes	Theme 1: Existing information on POP-BFRs Sub-themes: • Import quantities • Regulatory status • POP-BFRs production status • POP-BFRs uses *Data source: Production-regulatory-advocacy group*	Theme 2: Gaps in POP-BFRs information flow Sub-themes: • Insufficient POP-BFRs data and information • Communication breakdown Theme 3: Barriers to generating and sharing POP-BFRs information Sub-themes: • Technical difficulties • Rarely updated and barely used channels *Data source: Production-regulatory-advocacy group and* *consumer group*	Theme 4: Consumers’ insights into CiP information access and sources Sub-themes: • Ways in which consumers seek and access CiP information Labeling concerns: clarity and trust *Data source: Consumer group*
	Theme 5: Optimizing POP-BFRs information availability and accessibility Sub-themes: • Regulation as an enabling factor • Integrating CiP information into maternal and child health services • Maximizing CiP information access with multiple channels • Using consumer awareness to drive for transparency *Data source: Consumer group and* *production-regulatory-advocacy group*		

The following sections provide a detailed examination of the themes, and their corresponding subthemes outlined in Table 3.

#### Theme 1: Existing Information on POP-BFRs

Participants in the industry-regulation-advocacy group cited various types of existing information about POP-BFRs. Government officials had estimates of the quantities of pure POP-BFRs imported into the country: “*So, in 2019, there were over two tons of pentabrodiphenyl ethers. Then there’s also the octabromodiphenyl ether*” (Government official G2). This information was connected to their phase-out under South Africa’s POPs regulation, which came into effect in 2019,^57^ and the prohibition implemented in 2021. They surveyed the quantity of POP-BFR chemical formulations imported into the country before these legislations were enacted (Government officials G1 and G2).

A plastics manufacturer provided information about the uses of POP-BFRs in electronics and wire cables in South Africa. Also acknowledged that the occurrences of legacy POP-BFRs contaminants in children’s plastic products are linked to WEEE recycled materials. Additionally, the participant noted that products such as toys were not being manufactured in South Africa: “*The brominated flame retardants are used in South Africa predominantly in electrical cables, and a little bit is used in the electronics industry. So, if it ends up in consumer or baby products, the chances are it’s in recycled plastic. We hardly manufacture any plastic toys in South Africa. The toy industry has died”* (Plastics producer PI1).

A flame retardant manufacturer provided information indicating that POP-BFRs have not been locally manufactured since implementing the POPs regulation. The participant also suggested that the flame retardants produced locally were not among those restricted by law and were intended for applications in buildings and tents: “*Our current flame retardants are halogen-free and do not contain fluorine, chlorine, bromine, iodine, or anything that releases dangerous substances”* (Flame retardants manufacturer CI1).

#### Theme 2: Gaps in POP-BFRs Information Flow

##### Sub-Theme: Insufficient POP-BFRs Data and Information

Participants in the industry-regulation-advocacy group stated they had insufficient information about POP-BFRs. The government officials lacked data on which consumer products, including children’s plastic products, contained POP-BFRs (Government officials G3, G4). They emphasized that the lack of information significantly affected the implementation of risk reduction measures. Similarly, there was data on the content and percentage of products containing POP-BFRs entering the waste stream (Waste manager EW2).

The interviewed NGO representatives raised concerns that the lack of data impacted their activism and awareness-raising campaigns: “*Well, the information that should be available concerning these products is generally lacking, and* that is *really a problem. The public appetite for this information is massive. So, if we don’t have the proper structures in place now to manage the information or the flow of information about those chemicals, then we will never be able to do it”* (NGO representatives NG1, NG2).

The consumer group participants lacked information regarding POP-BFRs: *“No, I don’t have information about those (POP-BFRs) chemicals. And I feel sort of helpless when it comes to toys, like I don’t have control over it”* (Consumer PP28). Most participants did not recall seeing information on flame retardants in general on product labels: “*I do not think there is anything that actually kind of stands out on the label that would inform the general public that there are flame retardants and that it is something potentially harmful” (Consumer P1).* Others cited the lack of product labeling: “*When our kids were babies, the BPA was something we were quite conscious of. We were also very concerned about lead. So, we were always checking labels. But, often, the labels weren’t there, or there was very little information. So that was a particular concern for us. We never came across flame retardants. But yeah, the information is just not always there” (*Consumer P3).

The consumer group participants were worried that the lack of information prevented them from taking precautions and making informed decisions about which products to purchase for their children: *“. . .so we know that we need to be more cautious about which product to buy, but if they’re not labeled, how do we know what’s risky and what’s not?”* (Consumer P1). Another participant in the consumer group highlighted the importance of clear warnings on product labels and packaging materials regarding chemicals such as POP-BFRs: “*I want clear and noticeable warning about chemicals used in a product indicated on the labels of the toys” (*Consumer P18).

##### Sub-Theme: Communication Breakdown

Participants in the industry-regulation-advocacy group reported experiencing communication breakdowns due to the lack of access and unresponsiveness of stakeholders in the supply chain. For instance, government officials noted that they could not obtain CiP information from product manufacturers, particularly for items not produced in South Africa and imported from abroad (Government officials G1, G3).

Additionally, government officials reported experiencing difficulties with stakeholders in South Africa. In one scenario, officials from one governmental department knew the quantities of POP-BFRs imported into the country. However, they did not have access to information about the chemical importers or end-users as that information was hosted in another department: “*We should know from their website, but they do not specify the users, basically. So, it is classified information. We will not know the importers as well. It is all classified information”* (Government official G2).

In another scenario, government officials were unaware of local POP-BFRs uses due reluctance of the industry in sharing the information: *“Following registration, we were supposed to get the list of the users from that process because the registration means, “Okay, I am here, I am still using this chemical and here is the phase-out plan that I am going to implement from now until the end. It was supposed to happen three months after the publication of the regulation. It has been nine months. We have not received anything. So, we will not know who’s using it because nobody registered”* (Government official G1). They assumed the industry hesitated to share information about legally restricted chemicals for fear of potential consequences. *“We went to the chemical industries, we requested information, and they never came back to us. Even those that we suspect could have the information we need. Perhaps others are aware that these chemicals are listed in the Stockholm Convention, so they may not be forthcoming.* That is *the unfortunate part” (*Government official G3).

#### Theme 3: Barriers to Generating and Sharing POP-BFRs Information

##### Sub-Theme: Technical Difficulties

Participants in the industry-regulation-advocacy group attributed the lack of data and information (**theme 2**) to several factors. The government can generate information and data from product monitoring and surveillance activities as a regulatory body. However, government officials identified challenges in conducting product monitoring activities, such as inadequate capacity to test for POP-BFRs in children’s products, primarily due to technical difficulties and cost implications: *“The main challenge, at the moment, it’s the lack of capacity on our side. We banned these chemicals. However, for POP-BFRs that are in products, we really don’t have the resources to test those products. We can regulate this, but we are skeptical about trying to regulate something that we cannot enforce. So, we thought it might be better to sort ourselves out before publishing a regulation to ban chemicals in the products we can enforce”* (Government official G1).

Although they were not monitoring POP-BFRs in children’s plastic products at the time, they noted that the focus might shift in the future: *“We cannot focus on that right now; we cannot do that right now. Perhaps in the future, we will have the capacity to monitor and regulate these chemicals in products. So, for now, we are not doing much on that, basically”* (Government official G2). They also noted that research would inform their policy decisions and raise public awareness: *“We know a lot about the effects of these chemicals, but we want to research to know exactly which products are coming with these chemicals and where they are coming from. Let’s close this gap before we commit ourselves to taking further action on the products*” (Government official G1).

##### Sub-Theme: Rarely Updated and Barely Used Channels

Furthermore, government officials outlined their strategies for disseminating information about general chemical hazards to the public: “*We have a program on chemical awareness. Last year, we conducted public awareness workshops focusing on several aspects of chemical management. We gave a presentation on household chemicals and focused a bit on flame retardants”* (Government official G2). Facebook social media was also used: *“We have a Facebook page. It’s basically on chemicals management. We talk about things like flame retardants, and so forth. We encourage people to join the Facebook page on chemicals management”* (Government official G2).

However, the Facebook page had not been updated since the Covid-19 lockdown, and only a small number of people engaged with the page: “*But since the lockdown, we haven’t been updating, and we haven’t been posting anything. That page is not active” (Government officials G2, G3).* Also, the workshops were not being conducted regularly: *“We are not actively sharing information even though the Stockholm Convention requires us to communicate information to the public, especially regarding the hazardousness of POPs”* (Government official G3).

On the other hand, government officials communicated with the chemical industry through a special committee comprising industry, academia, and NGO representatives. They have been sharing information, such as the status of POP-BFRs regulation. The POP-BFRs regulations were also published in the government gazette for industry access. However, they were unsure whether the information reached all the committee members (Government officials G2, G4, G5).

#### Theme 4: Consumers’ Insights Into CiP Information Sources and Access

While participants in the industry-regulation-advocacy group shared their methods for generating and sharing CiP information, including the challenges faced (**theme 3)**, participants in the consumer group, as recipients of the information, reported how they sought and accessed the CiP information. They also expressed their concerns about their primary source of information.

##### Sub-Theme: Ways in Which Consumers Seek and Access CiP Information

Twenty-six (59%) participants in the consumer group reported product labels as their source of information about the chemicals in children’s products. Only 7 (16%) participants reported other sources of information, including the internet, parenting newsletters, and podcasts: “*I’ve definitely read online and also* [listened to] *a podcast. I recall hearing on a podcast that we no longer have BPA in many plastic products, but there are numerous other ingredients that may be even more toxic*” (Consumer P5).

One participant indicated they sought information from peer parents and health professionals: “*I think that on the whole, for this kind of information, I get it from sort of conversations with others, which might from time to time spark my looking up more on the internet. If you are in a sort of nursery school kind of thing, you tend to talk to the other parents that you meet and come to some sort of a view on what’s good and what’s bad for your kids through those conversations*” (Consumer P4).

Most consumer participants (98%) rarely looked for information specifically about POP-BFRs: “*In terms of toys, I don’t recall ever checking for those chemicals*” (Consumer P5). However, a few searched for information about chemicals such as lead and bisphenol A (BPA): “*For plastic stuff, I just look for BPA-free. Well, in fact, I then moved away from plastic entirely and got metal and silicone bottles*” (Participant P4, P5). Some also checked safety warnings and the product’s origin information (Consumers P11, P23, P26). Interestingly, one participant assumed that children’s products are free of harmful substances and therefore did not seek information about chemicals: “*I usually look at how a product helps with each stage of the child’s growth. I never considered reading about the chemicals because I assumed they are made with the best care*” (Consumer PP32).

##### Sub-Theme: Labeling Concerns: Clarity and Trust

Several challenges related to product labels were reported. Some noted that the labels can be unclear and difficult to understand: *“I think in general the label is misleading consumers as there is information, yet it is not always in an understandable form, and not large enough to gain the attention required”* (Consumer PP1). “*It depends on the company manufacturing; some companies are vague about the production of their products; they will not list the ingredients and warnings*” *(*Consumer PP24). Another participant felt that the information on labels is untrustworthy due to the lack of regulation on consumer product labeling: *“I have very low levels of trust in information provided by many manufacturers given my knowledge as a lawyer that there is a lack of regulation and enforcement in this area”* (Consumer PP30).

One participant compared the quality of labels from different countries, echoing how regulations influence labeling practices: “*And to be honest, it is usually your Chinese toys. I am sorry. The labels are often poorly worded or fail to list all the necessary information. Or the translation’s really poor. I find that toys from the EU (European Union) tend to be more informative in terms of labeling. But your toys from the East, I do struggle with. Also, it’s much harder to find information on toys from the East than it is for toys from the EU. And maybe that’s got to do with regulatory standards*” (Consumer P1). Another participant pointed out that regulations influence the overall safety of consumer products: “*I feel that if something has been produced for a European market or the American market, it is probably subjected to the safety regulations in those regions, and therefore it would be safer. Whereas some Chinese brand, I mean, that’s just an example, but any Asian brand, the chances that it contain these contaminants is high*” (Participant P3).

#### Theme 5: Optimizing POP-BFRs Information Availability and Accessibility

Participants in both study groups desired improved POP-BFRs information availability and access. They proposed several actions, including establishing legal requirements for information disclosure, integrating CiP information in maternal and child health services, and scaling up awareness-raising campaigns. Also, participants in the consumer group highlighted their preferences for receiving CiP information. This theme is explored under several sub-themes below.

##### Sub-Theme: Regulation as an Enabling Factor

Participants in the consumer group believed there should be legal requirements for product manufacturers, importers, and retailers to disclose POP-BFRs information for children’s plastic products placed on the market. They referred to BPA as an example of existing regulations and labeling requirements (Consumers P3, P4). They also believed that implementing a product certification system, for instance, by the South African Bureau of Standards, would ensure accurate disclosure of CiP information (Consumer P19).

##### Sub-Theme: Integrating CiP Information in Maternal and Child Health Services

The consumer participants also felt that ensuring CiP information is available and accessible during the prenatal and postnatal periods would have a significant impact. The prenatal and postnatal stages are critical for parents; they frequently attend clinics or seek health advice for their babies. The stages present a significant opportunity that should not be missed: *“I went for antenatal classes when I was pregnant with my boy. They provide information about the delivery process and what happens to your body. One thing I can tell you is that this is the most underutilized time to educate mothers about the dangers of these chemicals on children”* (Consumer P2).

Another participant echoed this view and added that clinic health professionals should be equipped with resources and CiP information on POP-BFRs to educate parents attending clinics: “*Not all people attend antenatal classes, but the one thing that absolutely everybody does is take their baby to the clinic. So, if clinic sisters had resources that they could share with parents, then you could reach almost 100%. Only the very, very irresponsible mothers would not go to the clinic*” (Consumer P5).

Another consumer participant emphasized that CiP information should be part of the public health promotion agenda: *“When you are expecting a baby or have a baby, so much information comes to you from all sorts of sources. You talk to the clinic sister, the midwife, or the gynecologist. We have been very successful at the breastfeeding message*, for example, *saying, breast milk is best. Every expectant mother hears this a million times. But no one tells us about the chemical contaminants that result from our daily life habits. So, there’s kind of an imbalance. I think the message about these chemicals in products could be part of our public health discourse*” (Consumer P3).

A suggestion to include CiP information in the “Road to Health” booklet was also given: “When your child is born, regardless of what hospital the child is born in, you get the Road to Health booklet, and it’s issued by the Department of Health. It’s like a health passport almost. And every time you go to the clinic, you’re supposed to take that book with you, and they record the child’s weight and height and things like that. . . so that booklet could be another place to add information, just one page saying, “Did you know that the ingredients of food, skin products, toys, fabrics, can impact the health of your child. Look out for. . .” And then you sort of have a list of 5 or 10 dangerous chemicals” (Consumer P5).

##### Sub-Theme: Maximize CiP Information Access Through Multiple Channels

Channels such as social media, news outlets, and newsletters were highly preferred by participants in the consumer group (Consumers P5, P9, P13, P28, P33, P39). Other participants in the same group suggested using influencers: *’A lot of moms read parenting books. So, I think if these mom influencers write about flame retardants and products that might have them, then that would help. I think it could certainly change some behaviors*” (Consumer P4), and digital platforms such as a website and mobile applications with barcode readers: “*I would say it’s important to not just have one source of information; I would say that there should be a consumer website with information about products and these chemicals. There are apps that you can use when you’re doing grocery shopping that allow you to scan the barcode, and then it will give you product information and ingredient list*” (Consumer P5).

##### Sub-Theme: Leveraging Consumer Awareness to Drive for Transparency

The interviewed NGO representatives contended that if the government invested in raising awareness about POP-BFRs and their health risks, it would increase consumers’ understanding of the problem and create a desire to demand CiP information from product manufacturers: “*There must be awareness raising by the government. I think once the public has a sense of what is happening, to an extent, they will make their own decisions about how they enter the debate and how they contribute to the debate. I just think that consumers are just so unaware, especially with children’s products. And I think there will be a huge demand for that information from there*” (NGO representative NG2). However, they acknowledged that it may take time before the impact of these initiatives is noticeable: “*But it took years and years and years to get to that point where consumers are aware of BPA* for example, *and it’s the consumer demand that made it necessary for that shift in legislation and mindset*” (NGO representative NG1).

## Discussion

**Figure 2.**
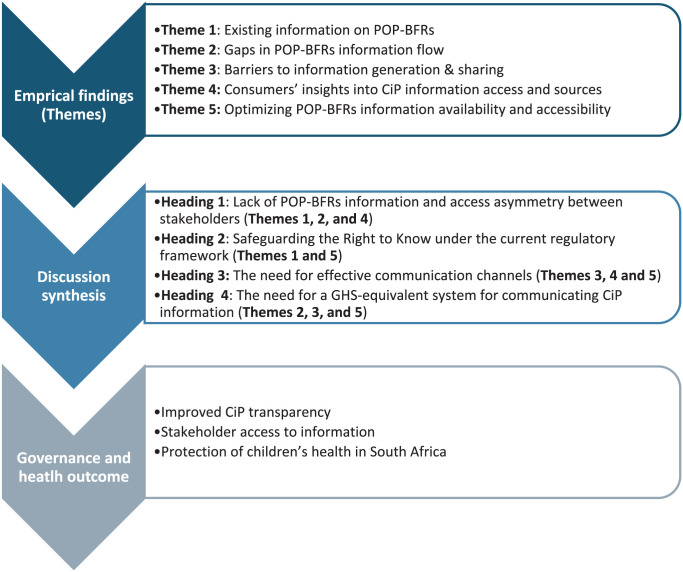
The figure illustrates how the empirical findings derived from the study themes inform the main discussion areas.

This discussion section integrates the study findings to provide a holistic understanding of the current state of CiP information on POP-BFRs in children’s plastic products within the South African context. Drawing on the themes identified in the results section, the discussion examines how existing information, systemic gaps, and barriers to information exchange collectively shape stakeholders’ access to CiP information. It progresses from identifying information asymmetries to examining the need for effective communication mechanisms and the development of a standardized system for CiP disclosure, as illustrated in Figure 2.

### Lack of POP-BFRs Information and Access Asymmetry Between Stakeholders

Our study examined the availability and accessibility of CiP information on POP-BFRs in children’s plastic products among stakeholders in South Africa. We found that information on POP-BFRs in children’s plastic products was mainly lacking. Stakeholders responsible for regulation and advocacy had limited access to general information on POP-BFRs and the potential risks they pose to consumers, while consumers had no access. These findings underscore the need for stronger regulatory measures to improve access to and availability of CiP information on legacy POP-BFRs.

Our findings align with those reported in earlier global studies. Dannwolf et al^41^ discovered a lack of CiP information about IAS and NIAS in toys in a case study conducted for the UNEP’S CiP program. Similarly, regulatory stakeholders in the building materials supply chain case study reported receiving inadequate and vague CiP information from manufacturers.^43^ This has also been observed in the electronics^42^ and the textile product sectors.^44^ A recent comprehensive review of available global databases on CiP information revealed a lack of data for IAS and NIAS for various products across different jurisdictions, suggesting a persistent global concern.^32^

The lack of information on NIAS, such as POP-BFRs, in children’s plastic products can be linked to the complexity of the product supply chain. The increasing demand for cheaper products pushes manufacturers to use recycled plastics, like those from WEEE, as raw materials to lower costs.^58^ However, these materials are often not screened before reuse to determine their chemical makeup regarding IAS and NIAS based on their original use and the recycling process. Such analyses are expensive and are viewed as not cost-effective by manufacturers.^32^

Furthermore, transparency in recycling streams is limited, making it hard for stakeholders outside the supply chain to track the chemical content of recycled plastics.^59^ As a result, whether virgin or recycled plastics are used in children’s products, manufacturers may lack data on the presence of POP-BFRs in raw materials. Even when manufacturers know the chemical content of products, especially for hazardous chemicals, they may deliberately obscure it with vague terms. Additionally, industry actors are often hesitant to disclose information on POP-BFRs after their listing in the Stockholm Convention due to fears of regulatory repercussions, as the study findings show. Consequently, the CiP information might not reach consumers and regulatory authorities.

Our findings also suggest that there is no CiP information exchange between product manufacturers in the country of origin and end-users of the products in importing countries. South Africa, for example, imports approximately 90% of its plastic toys from China^26^ due to the increasing demand for affordable products. These imported products from large producers are typically cheaper than locally made ones, benefiting from economies of scale.

### Safeguarding the Right to Know Under the Current Regulatory Framework

The absence of CiP information on POP-BFRs in children’s plastic products infringes on the South African consumer’s right to know, a vital human right. Therefore, establishing mandatory disclosure and product labeling is essential to promote transparency.^60^ This means that for CiP information on POP-BFRs in products to be available, regulations must require that the manufacturing industry generate and share this information with all relevant stakeholders in the supply chain. The European Union’s (EU) Registration, Evaluation, Authorization, and Restriction of Chemicals (REACH) regulation is a prominent example of best practice in this aspect. Under REACH, product suppliers must provide CiP information when their products contain substances of very high concern (SVHC) at a concentration above 0.1% by weight.^61^ This information is then submitted to the substances of concern in a products database (SCIP; https://echa.europa.eu/scip) accessible to consumers. Therefore, it empowers consumers by enabling them to exercise their right to know about SVHC in the products they purchase. Apart from SCIP, the Digital Product Passport (DPP) under the Ecodesign for Sustainable Products Regulation (ESPR) is another initiative for ensuring information about a product is available throughout its life cycle.^62^

In contrast, South Africa lacks a comprehensive approach to CiP information similar to those under REACH and ESPR. For instance, the Consumer Protection Act (CPA) of South Africa does not explicitly define the right to know about the chemical content of products. While it emphasizes general product safety, it does not mandate the disclosure of hazardous chemicals.^63^ Therefore, an overarching chemical legislation is urgently needed to align South Africa with international best practices such as the SCIP and DPP. Such legislation could provide a foundation for establishing a chemical register for tracking chemicals, including those in consumer products. It could also introduce full disclosure requirements for relevant industry sectors, thereby enabling transparency and protecting consumers’ right to know.

### The Need for Effective Communication Channels

Our study found that the use of ineffective channels was one1 of the challenges to the accessibility of CiP information. For example, regulatory stakeholders relied on Facebook to communicate about chemical risks with the public. However, none of the consumer group participants used Facebook as their source of CiP information. Additionally, while some participants in the consumer group did not habitually read the product labels, others found them difficult to understand.

Klaschka and Rother^64^ argue that a culture of engaging with labels, combined with prior experiences, the time invested in seeking information, and general awareness of hazards, can help consumers comprehend the information on those labels. However, Hartmann and Klaschka^50^ found that even knowledgeable consumers struggled to understand the CiP information on product labels. Nevertheless, understanding the information on the label and increased awareness about chemicals present in products can influence consumers’ purchasing, usage, and disposal of products.^49^ Therefore, communication channels must be accessible, clear, and understandable to ensure consumers’ access to information on POP-BFRs in children’s plastic products.

We also discovered that South African participating consumers lack trust in the information on product labels. A study conducted in South Korea suggests that consumers tend to trust the government as their source of information rather than product manufacturers.^65^ In contrast, consumers in Europe trust consumer associations, researchers, and scientists as sources of information.^66^ A study from Canada found that pregnant women trust prenatal classes as a source of information regarding chemicals in household products.^67^ Another study suggested that pregnant women feel confident receiving information from healthcare professionals.^68^ These findings suggest that bridging the trust gap is crucial for enhancing the accessibility of CiP information on POP-BFRs in children’s plastic products.

In our study, participants were confident in and trusted maternal and child health services as a source of information. Furthermore, we discovered that participants trust South Africa’s “Road to Health” book, a health record and education booklet produced by the National Department of Health (NDoH) as part of child health initiatives. It is provided free of charge to parents and caregivers postnatally. The NDoH would need a dedicated section on common hazardous chemicals such as POP-BFRs in children’s products and a guide for safer products in the Road to Health booklet. Since the booklet is a trusted resource, it would vastly improve parents’ and caregivers’ access to CiP information.

Integrating CiP information in maternal and child health services may face some challenges. For instance, healthcare professionals often lack adequate training on chemical exposure risks, leaving them feeling unprepared and under-informed on the topic.^69,70^ Therefore, they may be unable to advise patients about ways to avoid chemical exposure and minimize health risks. Healthcare professionals must be trained in identifying and mitigating chemical exposure risks to educate patients effectively.

The use of smartphone applications with barcode scanners for sharing and accessing CiP information on POP-BFRs was recommended by participants in our study. These tools have been implemented before. For example, the European Chemicals Agency (ECHA) developed a smartphone application called “Scan4Chem,” (https://www.askreach.eu/app/) which allows consumers in 21 European countries to check whether SVHC are present in products. Consumers can also request the CiP information directly from product suppliers.^71^ A similar application, “ToxFox, ” (https://www.bund.net/themen/chemie/toxfox/) was launched in Germany.^72^ However, mobile applications face their own set of challenges.

A European study focusing on consumers’ behaviors toward smartphone applications revealed that users do not engage with these applications regularly. The inconvenience of waiting for suppliers’ responses, particularly in the case of “Scan4Chem,” was cited as a reason for inconsistent use.^73^ Additionally, access to smartphones and a certain level of digital literacy are needed to utilize this application effectively. Furthermore, the design of such applications must be easy to navigate to encourage usage. For similar applications to be used for CiP information on POP-BFRs in South Africa, further studies would be necessary to assess their effectiveness locally.

### The Need for a GHS-Equivalent System for Communicating CiP Information

Our findings on the unavailability and inaccessibility of information on POP-BFRs in children’s products can be attributed to the gaps in the current UNEP’s CiP program guideline,^36^ as the only global framework for CiP information. One of its significant gaps is the lack of a standardized system for communicating CiP information, which may result in stakeholders’ differing implementations across various countries and stages of the product life cycle. This situation can result in inconsistencies and variations in CiP information or a lack of such information altogether. It underscores the need for a global, standardized system for communicating CiP information.

An example of a standardized system is the Globally Harmonized System of Classification and Labeling of Chemicals (GHS).^74^ The GHS focuses on chemical substances and mixtures and provides a standardized format for classification and communicating chemical hazards through labels and safety data sheets.^74^ For example, under GHS, chemical labels must contain pictograms conveying hazard information, signal words, hazard and precautionary statements, and product and supplier identities. This approach ensures that consistent information is provided throughout the chemical life cycle. Therefore, there is a need for a GHS-equivalent system with standardized criteria for classifying chemicals in consumer products and respective labeling formats for those products.

The labeling criteria should include essential elements, such as precautionary and hazard statements, to convey the CiP information to product consumers and other stakeholders. For example, labels on children’s plastic products could include statements such as “contains recycled plastics” or “may contain cancer-causing chemicals” when, for example, WEEE recycled plastics have been used. Since plastic products, such as toys, often contain a mixture of chemicals, the classification should be based on the most severe chemical hazards. With this standardized system, manufacturers would need to classify and label their products based on the health hazards posed by the chemicals those products contain. For this standardized system to be successful, countries would need to integrate it into their existing regulatory frameworks, much like the current implementation of the GHS. Therefore, the globally standardized system for CiP information would facilitate communication between various stakeholders and stages of the product life cycle. Additionally, it would help to fill the existing CiP information gaps concerning POP-BFRs in children’s plastic products, particularly in countries such as South Africa, which lack a regulatory framework for CiP information but still rely on imported products.

### Study Strength and Limitation

The study’s overall strength lies in its comprehensive insights from diverse stakeholders across the life cycle of children’s plastic products. Also, unlike previous research, which has generally addressed chemicals in a broad context, this study focused on a specific group of chemicals, POP-BFRs. Furthermore, it examined the entire product life cycle, making it unique. The findings provide valuable insights that can inform further discussions to enhance the availability and accessibility of CiP information on POP-BFRs and other hazardous chemicals nationally and internationally.

However, the study has some limitations. Firstly, the consumer study group primarily consisted of university-educated parents and caretakers due to the COVID-19 lockdown restrictions. With this high level of education, their understanding, perceptions, and behaviors may not accurately reflect those of the general population. Also, the consumer group may reflect the views of Cape Town residents and not the broader South African population. Furthermore, insights from the product manufacturers are not included in this study. Future studies should aim to address these gaps.

## Conclusion and Recommendations

Considering children’s unique vulnerability, daily-use products that expose them to banned hazardous chemicals like POP-BFRs present a significant health concern. Strong regulatory actions are needed to address this threat and disclosure of information about the presence of these contaminants in children’s products is essential to help stakeholders make informed decisions. Yet, despite global frameworks for CiP information, such as the old SAICM and the new GFC, our findings reveal considerable gaps in the availability and accessibility of information on POP-BFRs in children’s plastic products in South Africa. While our study specifically assessed information on POP-BFRs in children’s plastic products, the results suggest similar gaps may be prevalent for IAS and other NIAS, and in a wider range of consumer products. Our findings underscore the need for national and international coordinated efforts.

To address the information gaps nationwide, the South African government, through the Department of Forestry, Fisheries, and Environment, in collaboration with the Health Department and the National Consumer Commission, should develop and implement comprehensive chemical legislation, establish a national chemical register, and require full disclosure of CiP by industry sectors. Also, strengthening evidence base through analytical monitoring of POP-BFRs along the product life cycle is essential. Such evidence would complement the need for CiP disclosure and help regulators prioritize interventions. Additionally, the relevant departments should launch public awareness initiatives and invest in CiP communication channels tailored to the local context.

At the global level, UNEP should create a standardized system for sharing CiP information across global supply chains within the 2023 GFC. Once established, South Africa and other countries should incorporate it into their national laws. These measures would mark significant progress towards ensuring that CiP information on POP-BFRs in children’s plastic products is available and accessible to relevant stakeholders worldwide and nationally. This advancement would promote chemical transparency in consumer products, uphold consumers’ right to know, and protect the vulnerable population.
